# Glycosylation Pattern of Serum Clusterin in Psoriatic Arthritis and Rheumatoid Arthritis—The Search for New Diagnostic Glycomarkers

**DOI:** 10.3390/ijms252313060

**Published:** 2024-12-05

**Authors:** Ewa Maria Kratz, Monika Kacperczyk, Izabela Kokot, Agnieszka Piwowar, Bogusława Konopska, Renata Sokolik, Lucyna Korman

**Affiliations:** 1 Division of Laboratory Diagnostics, Department of Laboratory Diagnostics, Faculty of Pharmacy, Wroclaw Medical University, Borowska Street 211A, 50-556 Wroclaw, Poland; m.b.kacperczyk@gmail.com (M.K.); izabela.kokot@umw.edu.pl (I.K.); boguslawa.konopska@umw.edu.pl (B.K.); 2 Department of Toxicology, Faculty of Pharmacy, Wroclaw Medical University, Borowska Street 211, 50-556 Wroclaw, Poland; agnieszka.piwowar@umw.edu.pl; 3 Department of Rheumatology and Internal Medicine, Wroclaw Medical University, Borowska Street 213, 50-556 Wroclaw, Poland; renata.sokolik@umw.edu.pl (R.S.); lucyna.korman@umw.edu.pl (L.K.)

**Keywords:** clusterin glycosylation, psoriatic arthritis, rheumatoid arthritis, diagnostic markers

## Abstract

Psoriatic arthritis (PsA) and rheumatoid arthritis (RA) are connective tissue autoimmune diseases. The present study aimed to check whether serum clusterin (CLU) concentration and its glycosylation pattern may be markers differentiating these diseases—blood sera of patients with PsA (n = 37), RA (n = 34), and healthy subjects (control, n = 21) were examined. CLU concentration was measured using the ELISA test. Glycosylation was analyzed using lectin-ELISA with sialo-specific lectins from *Maackia amurensis* (MAA) and *Sambucus nigra* (SNA) recognizing sialic acid (SA) α2,3- and α2,6-linked, respectively, and fucose-specific lectins from *Lotus tetragonolobus* (LTA), *Ulex europaeus* (UEA), and *Lens culinaris* (LCA) specific to fucose α1,3-linked, α1,2-linked, and core fucose, respectively. Significantly higher CLU concentrations were observed in the PsA than in the RA patients. The expression of α2,6-linked SA was significantly higher in the PsA and RA patients than in the control. The expression of SNA-reactive SA was visibly higher in the PsA compared to the RA and control group but insignificant. Negative significant correlations between CLU concentrations and its glycans reactivity with LTA and UEA were also observed. Significantly higher serum CLU concentration, accompanied by a high expression of SNA-reactive SA and a reduced degree of Lewis^x^ and Lewis^y^ antennary fucosylation, may constitute a promising panel of parameters differentiating PsA from RA.

## 1. Introduction

In recent years, there has been an increasing prevalence of psoriatic arthritis (PsA) [1,2], which in Western countries has already reached 2–3% of the general population [3,4]. Psoriasis of the joints, like rheumatoid arthritis (RA), is an autoimmune, chronic inflammatory disease, characterized by a heterogeneous course, during which arthritis and osteoarthritis, nail dystrophy, or uveitis can occur [5]. There are no laboratory tests specific for PsA. As in most inflammatory diseases, acute phase reactants such as erythrocyte sedimentation rate (ESR) and C-reactive protein (CRP) may be elevated. However, a normal ESR and CRP should not be used to rule out a diagnosis of PsA, as these levels are increased in only about 40% of patients [6]. Rheumatoid factor and anti-cyclic citrullinated peptide antibodies are classically considered absent in the blood of psoriatic arthritis patients. A negative rheumatoid factor is considered a criterion for diagnosing PsA. However, results from various studies have shown a positive rheumatoid factor in about 2% to 10% of patients diagnosed with PsA, and approximately 5% are positive for anti-cyclic citrullinated peptide antibodies [7,8]. Johnson et al. [9] documented that antinuclear antibodies may also be positive in 14% of patients with PsA but usually at low titers. It should also be emphasized that psoriatic arthritis is a disease that affects the vast majority, although not always, of people with diagnosed psoriasis and manifesting psoriatic lesions on the nail plate or skin. This is a feature that distinguishes psoriatic arthritis from rheumatoid arthritis [10]. PsA should be differentiated from rheumatoid arthritis (RA). During PsA progression, a chronic inflammatory process occurs in the body, which most often involves the synovial membrane of the joints, leading to their destruction. Consequently, patients have to face pain and reduced quality of life, and, in some cases, even disability [11].

The differentiation between RA and PsA can be challenging for clinicians. The classifications commonly used to diagnose both diseases such as the Classification criteria for Psoriatic Arthritis (CASPAR) [12] and the 2010 European League Against Rheumatism (EULAR)/American College of Rheumatology (ACR) classification [13] cannot be used in every case, e.g., the CASPAR criteria set can be used with confidence in prospective trials; however, its performance when used retrospectively has not yet been assessed [14]. Physical examination is not enough to determine synovitis, tenosynovitis, or enthesitis, and, therefore, the CASPAR diagnostic capability is limited in psoriatic arthritis [15]. Additionally, many things may cause nail changes, including fungus, which is far more common than PsA [16]. Joints can also ache for various reasons such as osteoarthritis, gout, and fibromyalgia [17,18], and joint swelling or especially enthesis tenderness do not always reflect inflammation [19]. Considering the above information, it should be underlined that CASPAR is not a diagnostic criterion, it is a classification criterion. Therefore, being aware of the imperfections of the CASPAR criteria as a diagnostic tool in PsA, to improve the detectability of this disease, some researchers used Ultrasound-modified CASPAR criteria, which allowed for the identification of almost twice as many patients with PsA as when the conventional CASPAR criteria were used [19]. Moreover, symptoms may not be disease-specific in the early stages of psoriatic arthritis. It has been shown that in patients with PsA, a late treatment application, 6 months after the onset of symptoms, leads to significant joint changes, consequently affecting their physical performance [20]. Rheumatoid arthritis is characterized by an extensive degradation of the cartilage and underlying bone that causes suffering in many people worldwide [21]. The pivotal role of immune cell infiltration into the joint followed by bone erosions is among the most significant characteristics of RA [22]. It was documented that considerable permanent joint damage can occur within the first 2 years of disease development, and, thus, the optimal management of RA is essential during the first 3 to 6 months [23,24]. The same tests are mainly used to diagnose the joint diseases discussed. As no laboratory test is specific for the early stages of PsA and RA, new, reliable biomarkers are needed to enable early diagnosis and differentiation of PsA and RA, which will have an impact on the implementation of earlier, targeted, and, therefore, more effective treatment of both diseases. Our previous studies have shown that clusterin (CLU; also known as apolipoprotein J), which belongs to the glycoprotein family [25], may be taken into account as a promising biomarker of some diseases. In the context of psoriasis and RA diagnostics, the CLU concentration was also examined [26,27,28]. What important, protein glycosylation, including CLU, is one of the most common post-translational modifications, in which oligosaccharides are transferred to specific amino acid residues in proteins by glycosyltransferases. A growing body of evidence suggests that glycosylation is essential for various functional activities in organisms, such as the regulation of protein function, cell adhesion, and immune escape. Abnormal glycosylation patterns are closely associated with the development of several diseases, including cancer, inflammation, and autoimmune disorders. However, the underlying composition and structure of the glycosylated residues have not been completely determined for all glycoproteins. To date, investigations on the clinical applications of glycosylation have focused on sensitive and promising biomarkers, the development of more effective small molecule targeted drugs, and emerging vaccines [29]. Therefore, we decided to check whether CLU concentration in association with the variability of its glycosylation pattern could be used as an additional diagnostic marker allowing both diagnosis of PsA and RA, as well as differentiation of these diseases, which is not an easy task.

Clusterin is characterized by various activities and a propensity to interact with a wide spectrum of molecules and modulate a variety of molecular processes both in health and disease [30]. It can exist in secretory (sCLU) and nuclear (nCLU) forms. CLU is built from α and β subunits which, during enzymatic posttranslational processes, are glycosylated. To date, six N-glycan binding sites have been identified in the protein chain of the CLU molecule [25]. The mature form of clusterin is approximately one-third composed of carbohydrates [31], and variability in CLU glycosylation plays an important role in its involvement in physiological and pathophysiological processes [32]. The primary role of CLU is to act as an extracellular chaperone that prevents the aggregation of non-native proteins. It then binds to receptors on the cell surface, resulting in the internalization of the chaperone-cluster complex and subsequent lysosomal or proteasomal degradation [30]. The intracellular form of clusterin promotes the ubiquitination and proteasomal degradation of target proteins [33]. In addition, CLU is one of the inhibitors of the complement system, binds to all types of human immunoglobulins, and is involved in processes related to cell apoptosis [34]. These functions play an important role in the pathogenesis of PsA and RA, allowing us to suppose the involvement of CLU in these processes, making it useful as a diagnostic marker differentiating both diseases. What important, to date, clusterin was among the top 30 proteins included in a panel of protein biomarkers with the potential to predict response to biological therapy in psoriatic arthritis [35]. Some authors also indicated that clusterin concentration could serve as a potential predictive biomarker for disease activity and treatment response in early RA development [28]. Another important point demonstrated by Rohne et al. [36] is the relationship between changes in clusterin glycosylation and its function as a chaperone protein. Of the 43 identified CLU N-glycan variants, all are characterized by a complex structure containing either terminal sialic acid or galactose [37]. Additionally, almost half of them showed core fucosylation. Taking into account all the above information, we decided to analyze the profile and degree of clusterin sialylation and fucosylation in the sera of PsA and RA patients using a modified semi-quantitative lectin-ELISA test. The eventual changes in examined parameters were also taken into consideration as potential markers, the diagnostic utility of which was worth exploring.

## 2. Results

### 2.1. Clusterin Concentration

Clusterin concentrations were significantly lower in the sera of the RA patients (median value: 12.89 µg/mL) compared to the PsA group (median value: 20.50 µg/mL) with a significance of *p* = 0.000246. There were no other significant differences between the examined groups in CLU concentration. The median value of this parameter was 15.89 µg/mL for the control group of healthy subjects (Table 1).

**Figure 1:**
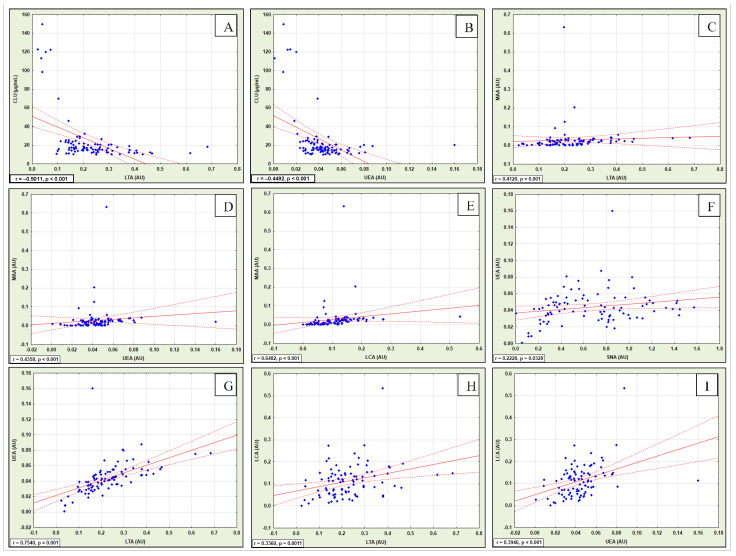
The correlations between examined parameters. Correlated parameters: CLU vs. LTA (**A**), CLU vs. UEA (**B**), MAA vs. LTA (**C**), MAA vs. UEA (**D**), MAA vs. LCA (**E**), UEA vs. SNA (**F**), UEA vs. LTA (**G**), LCA vs. LTA (**H**), and LCA vs. UEA (**I**). CLU—clusterin concentration in µg/mL; SNA—relative reactivity of blood serum CLU glycans with *Sambucus nigra* agglutinin; MAA—relative reactivity of blood serum CLU glycans with *Maackia amurensis* agglutinin; LTA—relative reactivity of blood serum CLU glycans with *Lotus tetragonolobus* agglutinin; UEA—relative reactivity of blood serum CLU glycans with *Ulex europaeus* agglutinin; LCA—relative reactivity of blood serum CLU glycans with *Lens culinaris* agglutinin. Specificities of lectins are presented in Section 4.3. Relative reactivities of CLU glycans with lectins are expressed in absorbance units (AU). The correlations were calculated using a Spearman’s rank test, and a two-tailed *p*-value (probability value) of less than 0.05 was considered significant. Blue diamonds—a set of points, each of which has the value of one variable defining the position on the horizontal axis and the value of a second variable defining the position on the vertical axis. Line of best fit (trend line) is marked in red. The red dashed line points to 95% of the confidence interval.

The Spearman’s rank correlations are shown in Table 2 and Figure 1A,B—significant negative correlations were found between clusterin concentrations vs. relative reactivities of CLU glycans with LTA (r = −0.5011, *p* < 0.001) and the relative reactivities of CLU glycans with UEA (r = −0.4492, *p* < 0.001).

### 2.2. Sialic Acids Expression in the Glycans of Serum Clusterin

The relative reactivities of CLU glycans with SNA were significantly higher in the RA (median value: 0.603 AU, *p* = 0.003711) and PsA patients (median value: 0.828 AU, *p* = 0.000186) in comparison to the control group (median value: 0.305 AU). There were no significant differences between the studied groups in the relative reactivities of CLU glycans with MAA (median values: control group: 0.020 AU, PsA group: 0.015 AU, RA group: 0.020 AU) (Table 1).

The relative reactivities of CLU glycans with MAA demonstrated significant positive correlations with the CLU reactivities with LTA (r = 0.4120, *p* < 0.001), UEA (r = 0.4358, *p* < 0.001), and LCA (r = 0.6482, *p* < 0.001) (Table 2, Figure 1C–E).

### 2.3. Fucose Expression in the Glycans of Serum Clusterin

No significant differences were found between the examined groups in the relative reactivities of CLU glycans with LTA. The median value of this parameter was 0.200 AU, 0.194 AU, and 0.252 AU for the control group of healthy subjects and the PsA and RA patients, respectively. The median value of relative reactivities of CLU glycans with UEA was 0.038 AU in the control group, 0.042 AU in the PsA group, and 0.046 AU in the RA group. The median values of the relative reactivities of CLU glycans with LCA in the control group, PsA, and RA groups were as follows: 0.078 AU, 0.120 AU, and 0.113 AU, respectively (Table 1).

There were significant positive correlations between the relative reactivities of CLU glycans with UEA vs. SNA (r = 0.2228, *p* = 0.0328) and LTA (r = 0.7540, *p* < 0.001). Significant positive correlations were found between the relative reactivities of CLU glycans with LCA and LTA (r = 0.3360, *p* = 0.001). Moreover, significant positive correlations were also observed between the relative reactivities of CLU glycans with LCA and UEA (r = 0.3946, *p* < 0.001) (see Figure 1F–I).

### 2.4. ROC Curve Analysis

**Figure 2:**
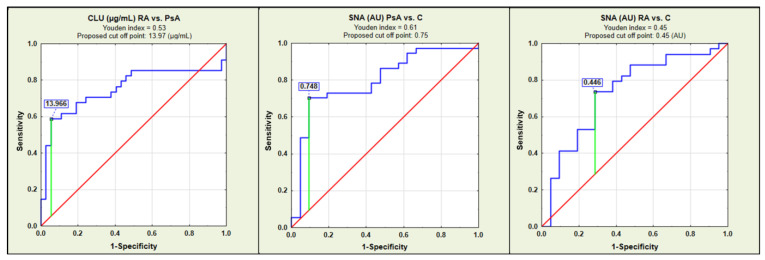
Receiver operating characteristic (ROC) curves for CLU concentrations and relative reactivities of blood serum CLU glycans with lectins for which the area under the curve (AUC) was higher than 0.700. C—control; PsA—psoriatic arthritis; RA—rheumatoid arthritis; CLU—clusterin concentration in µg/mL; SNA—relative reactivity of blood serum CLU glycans with *Sambucus nigra* agglutinin. SNA specificity is presented in Section 4.3. Relative reactivities of CLU glycans with lectin are expressed in absorbance units (AU). The reference line is marked in red, the receiver operating characteristics for an analyzed parameter in blue, and the cut-off point in green.

The results of ROC curve analysis and ROC curves for parameters with moderate clinical values (AUC > 0.700) are presented in Table 3 and Figure 2, respectively.

## 3. Discussion

PsA and RA should be considered chronic inflammatory diseases with overlapping features, which creates the need to search for new diagnostic markers specific to each of them. To meet these needs, in our previous studies using ATR-FTIR (attenuated total reflection Fourier-transform infrared) spectroscopic techniques, carried out in the same groups of patients with PsA and RA, we focused on selecting specific blood serum parameters and characteristic spectral features for each of the three studied groups, PsA, RA, and the healthy subjects, and we presented the results of discriminant analysis based on ATR-FTIR spectra, differentiating not only patients with PsA or RA from healthy individuals but also allowing for reliable differentiation of these two rheumatic diseases [38].

Clusterin concentration levels and the changes in its sialylation and fucosylation in the development of many diseases, including those of inflammatory background, have become a focus of the interest of scientists, including ourselves, as reported in previous articles. This glycoprotein interested us because of its multidirectional biological activity, which is also determined by the variability of its glycosylation [32]. We were curious whether, in the course of PsA and RA, the concentration of CLU and the pattern of its glycosylation would enable the differentiation of both diseases. To the best of our knowledge, there is no information on the variability of the CLU glycosylation pattern in the context of the development and progression of PsA and RA, and this is also the first research in which serum CLU levels were investigated in patients with PsA. To achieve the set goals, in the present study, we examined whether there are differences between the blood serum clusterin concentration and its glycosylation pattern between patients with PsA, RA, and the control group of healthy subjects. The significantly higher clusterin concentrations in PsA patients in comparison to the RA group, in our opinion, is an especially important observation because each additional parameter enabling the differentiation of PsA and RA is very valuable from the diagnostic and clinical point of view as well. The results of the ROC curve analysis additionally confirmed that CLU concentration can be taken into account as a parameter distinguishing PsA and RA patients with moderate clinical value (AUC = 0.754) with a sensitivity of 58.8%, specificity of 94.6%, and cut-off point of 13.97 µg/mL. Although the sensitivity shown in this analysis is not fully satisfactory, we believe that CLU concentrations are a promising additional marker for differentiating PsA and RA, the clinical usefulness of which should be verified in further, larger studies, also using other assay methods.

Studies conducted in patients with psoriasis, which in up to 85% of cases precede the onset of PsA [18], documented that serum clusterin levels were significantly higher in psoriasis patients compared to healthy controls [26,27]. Although the current study did not demonstrate any significant differences in serum CLU concentrations between the PsA patients and the control group, it is worth emphasizing that the median value of CLU concentrations in the PsA patients was visibly higher than the median calculated for the healthy subjects, which is consistent with the observations of Hashem et al. [26] and Holmannova et al. [27] for psoriasis patients. The hypothesized mechanism for this relationship is based on a compensatory and protective role that clusterin may play in psoriasis. As reported by Giang et al. [39], in psoriasis, there is the activation of the complement cascade with the formation of MAC (membrane attack complex; Cb5-9), against which clusterin exhibits binding properties, which may contribute to the prevention of cytolysis and cell damage [40]. In addition, clusterin is thought to be a chaperone protein that, under the conditions of oxidative stress associated with psoriasis, is involved in binding and removing cellular debris from the extracellular space, thereby preventing inflammation and autoimmunity [41]. Probably, similar mechanisms are activated in the development of PsA, but this hypothesis should be tested in further studies analyzing the interrelationships between CLU expression and the activation of individual complement components and MAC formation. On the other hand, Kropáčková et al. [28], based on the results of their study, suggested that increased serum concentrations of clusterin in treatment-naïve patients with early rheumatoid arthritis may serve as a predictive biomarker of disease activity and treatment response, which is very promising from the patients’ point of view and is worth further study for confirmation or exclusion of this observation. The fact that we did not show the presence of significant differences in serum CLU concentrations between RA patients and a group of healthy individuals may be due to several reasons. One of them may be the relatively lower number of participants in our study groups in comparison to these investigated by Kropáčková et al. [28], but the cause may also be changes in the molecular mechanisms associated with the synthesis of this glycoprotein, which may be influenced by the development of the disease but also by the implemented treatment or both of these factors. Considering the relatively small number of participants in the study groups and the fact that the PsA and RA patients had different treatment regimens in terms of medications used, and not all of them were treated for the same period, such comparisons could not be performed reliably in the current studies, although future studies should also take this aspect into account.

The pathogenesis of PsA and RA still hides many uncertainties. A potential reason for the significant difference in clusterin levels observed between PsA and RA patients may be IL-6, which plays an important role in RA development and progression. IL-6, one of the pro-inflammatory cytokines, drives together synovial inflammation and bone resorption through osteoclast activity [42]. McInnes et al. [43] observed the efficacy of IL-6 inhibitor treatment in reducing inflammation and structural damage of bones in RA. Moreover, some authors reported that IL-6 modulates CLU expression in colorectal cancer [44], which can also be applied to the pathogenesis of other inflammatory diseases, including RA. Although IL-6 production also occurs in PsA, it is not a specific marker, and it is possible that other cytokines, such as TNFα, IL-17, IL-22, and IL-23, play an important role in the disease development [45]. Our previous study documented that blood serum IL-6 levels do not differ significantly between PsA and RA, and IL-6 cannot be used as a marker differentiating these rheumatic diseases [38]. Additionally, no response to treatment with IL-6 inhibitors has been observed among PsA patients [46]. The exact role of CLU in these processes remains a puzzle and needs to be deciphered in future, broader studies.

The present study showed that the expression of terminal α2,6-linked sialic acid in serum CLU glycans significantly differentiates PsA and RA patients from the control group of healthy subjects, and this observation was confirmed by the results obtained in the ROC curve analysis, which showed that the expression of SNA-reactive sialic acid has a moderate clinical value (AUC > 0.700) to differentiate PsA and RA patients from healthy individuals (AUC = 0.798 and AUC = 0.735, respectively; proposed cut-off point was 0.748 AU and 0.446 AU, respectively) with sensitivity 70.3% and 70.6%, and specificity 90.5% and 71.4%, respectively. The presence of terminal sialic acid on glycoprotein glycans is known as a signal of inflammation development; thus, it was not a surprise for us that its higher expression is manifested in both rheumatoid diseases. It is worth emphasizing that, although increased expression of terminal sialic acid on serum CLU glycans is most likely associated with inflammation accompanying both PsA and RA, the sialylation profile of this serum glycoprotein differs depending on the factor causing inflammation, as exemplified by the CLU sialylation pattern observed by us in COVID-19 patients [47].

Interestingly, although we did not demonstrate any significant differences between the studied groups in the reactivity of CLU glycans with fucose-specific lectins, a moderate negative correlation between CLU concentrations and the expression of fucose, characteristic for Lewis^x^ and Lewis^y^ sugar structures, was observed. Taking into account the above, as well as the here previously discussed differences between PsA and RA vs. the control group in the expression of SNA-reactive sialic acid, it can be concluded that, in the course of PsA, high CLU concentrations, significantly higher than in RA, are accompanied by high expression of terminal α2,6-linked sialic acid with a simultaneous decrease/lack in the expression of antennary fucose of Lewis^x^ and Lewis^y^ oligosaccharide structures. Such a glycosylation pattern is observed for the CLU present in blood serum [25,48], the consequence of which is that CLU cannot bind via its glycans to the DC-SIGN receptor (dendritic cell-specific intercellular adhesion molecule-3-grabbing non-integrin), or such bonding is made difficult, which, in turn, disturbs or even inhibits removing complexes composed from clusterin and pathologically altered proteins which are addressed to the DCs (dendritic cells) via DC-SIGN [32,49]. Cunin et al. [41] documented that CLU binds specifically to late apoptotic cells but not to live, early apoptotic, or necrotic cells. Consequently, CLU potentiates, both in vitro and in vivo, the phagocytosis of late apoptotic cells by macrophages, remembering that fucosylated clusterin effectively interacts with macrophages by interacting with DC-SIGN. Moreover, the increased phagocytosis of late apoptotic cells induced by CLU favors the presentation and cross-presentation of apoptotic cell-associated antigens. The authors observed that, in a mouse model of apoptotic cell induced autoimmunity, and relative to control mice, CLU−/− mice develop symptoms of autoimmunity. The authors summarized that these results identify CLU as a new molecule involved in apoptotic cell efferocytosis and suggest a protective role for CLU in inflammation and autoimmune diseases. Considering the above observations of Cunin et al. [41], it can be assumed that in the course of autoimmune diseases such as PsA and RA, we are dealing with similar mechanisms of the anti-apoptotic action of CLU, and the elevated CLU concentrations in PsA accompanied by increased sialylation and reduced fucosylation of its glycans may impair CLU anti-apoptotic action.

Taking into account the multidirectional role of CLU glycosylation changes in the etiopathogenesis of many diseases, it can be concluded that enhanced sialylation together with decreased expression of fucose being a part of Lewis^x^/Lewis^y^ oligosaccharide structures is a marker of inflammation which is manifested in a variety of pathological conditions, regardless of the cause of their occurrence [32]. Sialylation of glycoproteins is involved in several pathophysiological processes. Sialic acids being a part of protein N-glycans prevent their interaction with the asialoglycoprotein receptor, thereby avoiding liver clearance [50]. In connection with the above, in the case of PsA development, does increased expression of terminal sialic acid on CLU glycans protect this glycoprotein from removal by the liver, thus enabling it to participate in other interaction pathways? Sialylation is also integral to several physiological functions such as protein conformation regulation, cell proliferation, migration, apoptosis, and cognitive processes [51]. It remains an open question what role the glycosylation pattern of serum CLU shown by us plays in the development of PsA and/or RA, especially since CLU has multidirectional effects which can be both positive and negative in terms of the development of a given disease. An example here may be the interaction with DC-SIGN receptor, with complement components, and pro- or anti-apoptotic action, which depend on the CLU glycosylation pattern. Further studies should also examine which of the interactions between CLU glycans, with special attention to sialic acid and fucose of Lewis^x^ and Lewis^y^ oligosaccharide structures, and their endogenous ligands may be the cause of the development of PsA and RA. The first step in this direction has been taken by us in the present study, because lectin-ELISA is a method that, through the reaction of glycans of the analyzed glycoproteins with lectins specific to them, to some extent mimics the accessibility of these glycans to their endogenous ligands in vivo.

In conclusion, a significantly higher serum CLU concentration, accompanied by a high expression of terminal α2,6-linked sialic acid and a reduced degree of Lewis^x^ and Lewis^y^ antennary fucosylation, may constitute a promising panel of parameters differentiating PsA from RA. In our opinion, more extensive studies, also using additional research methods, including a larger number of participants in homogeneous groups, inter alia in terms of the stage of the disease and the treatment used, with a similar percentage of women and men, aimed at explaining the molecular mechanisms in which CLU glycans participate, leading to the development and/or progression of PsA and RA, also in the context of the effectiveness of the applied treatment, seem to be an interesting and promising direction for further research.

## 4. Materials and Methods

### 4.1. Study Design and Sample Collection

Serum samples from recruited patients with PsA (n = 37, median age 42; 62% female) and patients with RA (n = 34, median age 48; 91% female) were collected at the Department of Rheumatology and Internal Medicine, Wroclaw Medical University, Poland. The diagnosis of RA was established according to the 2010 American College of Rheumatology/European League Against Rheumatism (ACR/EULAR) classification criteria for rheumatoid arthritis [13], while the Classification Criteria for Psoriatic Arthritis (CASPAR) were used to diagnose PsA [12]. For RA patients, the Disease Activity Score-28 (DAS28) scale was used to assess disease activity based on four components: the number of swollen and painful joints, serum CRP (C-reactive protein) concentration and ESR (erythrocyte sedimentation rate), and the overall assessment of the patient’s general health expressed on a visual analog scale. Based on the DAS28 scale, the degree of disease activity in RA patients can be determined as high (DAS28 > 5.1), moderate (3.2 < DAS28 ≤ 5.1), or low (DAS28 ≤ 3.2). PsA and RA patients were treated mainly by methotrexate and methylprednisolone but also by sulfasalazine, and, in some cases, the RA therapy was provided by hydroxychloroquine and leflunomide. Serum samples from recruited healthy individuals (C—control group, n = 21, median age 40, adequate gender distribution—81% female) were collected at the Department of Laboratory Diagnostics, Wroclaw Medical University. The BMI (Body Mass Index) of all participants was in the range of 21–28. This study was conducted in agreement with the Helsinki-II-declaration, and the protocol was approved by the Bioethics Human Research Committee of the Wroclaw Medical University (permission No. KB-549/2019, KB-707/2019, and KB-89/2023). Serum samples were obtained by peripheral blood collection, and, after 30 min coagulation, they were centrifuged at 1200× *g* for 10 min at room temperature. Samples were stored at −20 °C until examination. Written informed consent was obtained from all participants before this study.

### 4.2. Clusterin Concentration Measurement

Blood serum clusterin concentration was determined using the Human Clusterin ELISA Kit from Invitrogen (TermoFisher Scientific, catalog No. EHCLU; Frederick, MD, USA), according to the protocol provided by the manufacturer, without any modifications. The coefficients of variation (CV%) for the assay are defined by the manufacturer as <10% for intra-assay and <12% for inter-assay.

### 4.3. Lectin-ELISA

To determine sialic acid expression in glycans of serum clusterin (CLU sialylation), two biotinylated SA-specific lectins, *Sambucus nigra* agglutinin (SNA) and *Maackia amurensis* agglutinin (MAA), were applied. For the determination of fucose expression in CLU glycans (CLU fucosylation), three biotinylated fucose-specific lectins were used: *Lotus tetragonolobus* agglutinin (LTA), *Ulex europaeus* agglutinin (UEA), and *Lens culinaris* agglutinin (LCA). All the lectins were produced by Vector Laboratories Inc., Burlingame, CA, USA. The specificities of the lectins used are presented in Table 4.

It should be mentioned that the terminal α2,3-linked sialic acid limits the binding of LTA to α1,3-linked fucose of the Lewis^x^ oligosaccharide structure [53], and the presence of α1,2-linked fucose on the oligosaccharide antenna limits the attachment of α2,3-linked sialic acid to the terminal part of glycan [56]. Lectins can also react non-specifically with more than one oligosaccharide residue. The lectin-ELISA procedure according to Kratz et al. [57] and Janiszewska et al. [48] with the modifications described below was applied.

#### 4.3.1. ELISA-Plate Coating

ELISA plates (Nunc MaxiSorp, Thermo Fisher Scientific, Roskilde, Denmark) were coated with goat anti-human clusterin polyclonal antibodies (Invitrogen, Thermo Fisher Scientific, catalog No. PA1-26903; Rockford, IL, USA). The antibodies were diluted 1:10,000 for SNA and 1:5000 for MAA, LTA, UEA, and LCA in 10 mM TBS, pH = 8.5. After 2 h of incubation at 37 °C, the plate was washed three times with the same buffer. Next, because high absorbances of blank samples were observed in the preliminary experiments on the fucose reactivity of CLU glycans with LCA, oxidation of oligosaccharides present on the anti-human clusterin polyclonal antibodies coating the ELISA plate was performed. Sodium meta-periodate solution (100 mM NaIO4, 100 mM NaHCO_3_, pH = 8.1) was added, and, after 90 min incubation at room temperature in the dark, the plate was washed with 10 mM TBS pH = 7.5. For the other lectins, this step was not necessary. In the next common step, the free binding sites of the ELISA wells were blocked with 10 mM TBS, 0.1% Tween20, and 1% BSA (blocking buffer, pH = 7.5). After 2 h incubation at 37 °C, the plates were stored overnight at 4 °C.

#### 4.3.2. Sample Dilution

Sera samples were diluted in 10 mM TBS, 0.1% Tween20, pH = 7.5 to obtain a clusterin concentration of 50 ng/100 µL per well. Then, the samples deposited in the wells of the previously prepared microtiter plate were incubated at 37 °C for two hours. To minimize imprecision, each sample was analyzed in duplicate. Two pairs of blanks were added to each lectin-ELISA plate—these contained all reagents, but 10 mM TBS, 0.1% Tween20, pH = 7.5 (wash buffer) was used instead of patient samples—the same determination procedure was applied to the wells containing blank samples.

#### 4.3.3. Clusterin Reduction

To increase the availability of fucose present in CLU for lectins, a reduction step of clusterin bound with antibodies was included. For this purpose, dithiothreitol (DTT) was used, diluted in 0.1 M Tris-HCl, pH = 8.0 (2 mg/mL). After 70 min incubation at 37 °C, the plates were washed three times using 10 mM TBS, pH = 7.5.

#### 4.3.4. Lectin Interactions

The biotinylated lectins mentioned in Table 4 were used to detect the corresponding oligosaccharide residues. All lectins dilutions were established in the series of preliminary experiments using 10 mM TBS containing 1 mM CaCl_2_, 1 mM MgCl_2_ × 6H_2_O, 1 mM MnCl_2_ × 4H_2_O, 1% BSA, and 0.1% Tween20, pH = 7.5. *Sambucus nigra* agglutinin was diluted 1:2000, *Maackia amurensis* agglutinin 1:250, *Lotus tetragonolobus* lectin 1:1000, *Ulex europaeus* agglutinin 1:250, and *Lens culinaris* agglutinin 1:2000, and, next, plates were incubated for one hour at 37 °C.

#### 4.3.5. The Detection of Clusterin–Lectin Complexes

ExtrAvidin alkaline phosphatase labeled (Sigma-Aldrich, catalog No. E2636; Saint Louis, MO, USA) diluted 1:10,000 in the washing buffer was used to quantify complexes formed by clusterin glycans and lectins. After 1 h of incubation at 37 °C, the color reaction with disodium para-nitrophenyl phosphate was induced. The reaction was stopped with 100 µL of 1 mM NaOH per well, and, next, the absorbances were measured with a Mindray MR-96A Microplate Reader (Shenzhen Mindray BioMedical Electronics, Shenzhen, China) at 405 nm with a reference filter of 630 nm. The relative reactivities of CLU glycans with lectins were expressed in absorbance units (AU), after subtracting the absorbances of the blank samples.

### 4.4. Statistical Analysis

To perform the statistical analysis, Statistica 13.3PL software (StatSoft Inc., Tulsa, OK, USA) was used. The Shapiro–Wilk’s test was applied to analyze the normality of the distribution for the values of all parameters studied. The results obtained did not meet the normal distribution; therefore, nonparametric tests were used for statistical analysis. Using the Mann–Whitney U Test, the values of relative reactivities with lectins and CLU concentrations were compared between the study groups. The correlations between the values of the determined parameters were checked by Spearman’s rank correlation. *p*-values (probability values) < 0.05 were considered significant. Receiver operating characteristic (ROC) curves analysis was used to determine the diagnostic significance of relative reactivities of CLU glycans with sialo- and fucose-specific lectins, as well as levels of CLU. Based on the AUC, the clinical value of the laboratory test can be defined as 0–0.5—zero, 0.5–0.7—limited, 0.7–0.9—moderate, and >0.9—high [58].

## Figures and Tables

**Table 1:** The parameter values of blood serum CLU glycosylation analysis.

Parameter	Group		
	C n = 21	PsA n = 37	RA n = 34
	Mean ± SD Median (Range)	Mean ± SD Median (Range)	Mean ± SD Median (Range)
Clusterin concentration (µg/mL)	30.62 ± 37.30 15.89 (14.79–17.35)	21.36 ± 9.51 20.50 (16.69–24.01)	24.57 ± 32.36 12.89 (11.59–18.07) **^PsA^ *p* = 0.000246**
SNA (AU)	0.420 ± 0.316 0.305 (0.215–0.560)	0.837 ± 0.412 0.828 (0.387–1.157) **^C^ *p* = 0.000186**	0.624 ± 0.278 0.603 (0.423–0.851) **^C^ *p* = 0.003711**
MAA (AU)	0.064 ± 0.140 0.020 (0.006–0.056)	0.017 ± 0.013 0.015 (0.007–0.029)	0.020 ± 0.013 0.020 (0.008–0.031)
LTA (AU)	0.201 ± 0.101 0.200 (0.144–0.263)	0.205 ± 0.071 0.194 (0.163–0.229)	0.269 ± 0.150 0.252 (0.161–0.341)
UEA (AU)	0.037 ± 0.016 0.038 (0.028–0.048)	0.043 ± 0.012 0.042 (0.036–0.049)	0.049 ± 0.028 0.046 (0.034–0.058)
LCA (AU)	0.091 ± 0.059 0.078 (0.043–0.133)	0.128 ± 0.093 0.120 (0.066–0.161)	0.110 ± 0.057 0.113 (0.072–0.146)

C—control; PsA—psoriatic arthritis; RA—rheumatoid arthritis; SNA—relative reactivity of blood serum CLU glycans with *Sambucus nigra* agglutinin; MAA—relative reactivity of blood serum CLU glycans with *Maackia amurensis* agglutinin; LTA—relative reactivity of blood serum CLU glycans with *Lotus tetragonolobus* agglutinin; UEA—relative reactivity of blood serum CLU glycans with *Ulex europaeus* agglutinin; LCA—relative reactivity of blood serum CLU glycans with *Lens culinaris* agglutinin. Specificities of lectins are presented in Section 4.3. AU—absorbance unit. A two-tailed *p*-value (probability value) of less than 0.05 was considered significant (marked in bold).

**Table 2:** The correlations between values of analyzed blood serum parameters.

Compared Parameters	r	*p*
CLU and LTA	−0.5011	<0.001
CLU and UEA	−0.4492	<0.001
MAA and LTA	0.4120	<0.001
MAA and UEA	0.4358	<0.001
MAA and LCA	0.6482	<0.001
UEA and SNA	0.2228	0.0328
UEA and LTA	0.7540	<0.001
LCA and LTA	0.3360	0.0011
LCA and UEA	0.3946	<0.001

CLU—clusterin concentration in µg/mL; SNA—relative reactivity of blood serum CLU glycans with *Sambucus nigra* agglutinin; MAA—relative reactivity of blood serum CLU glycans with *Maackia amurensis* agglutinin; LTA—relative reactivity of blood serum CLU glycans with *Lotus tetragonolobus* agglutinin; UEA—relative reactivity of blood serum CLU glycans with *Ulex europaeus* agglutinin; LCA—relative reactivity of blood serum CLU glycans with *Lens culinaris* agglutinin. Specificities of lectins are presented in Section 4.3. Relative reactivities of CLU glycans with lectins are expressed in absorbance units (AU). r—Spearman’s rank coefficient. A two-tailed *p*-value (probability value) of less than 0.05 was considered significant.

**Table 3:** The results of receiver operating characteristic (ROC) curves analysis for relative reactivities of blood serum clusterin with lectins.

Parameter	Group		AUC	AUC with 95% Confidence Interval	Cut-Off Point	Sensitivity (%)	Specificity (%)	*p*
CLU	RA vs. PsA		0.754	0.629–0.878	13.97	58.8	94.6	0.0001
SNA	RA	vs. C	0.735	0.595–0.876	0.446	70.6	71.4	0.0010
	PsA		0.798	0.677–0.919	0.748	70.3	90.5	0.0000

AUC—area under the curve; C—control; PsA—psoriatic arthritis; RA—rheumatoid arthritis; CLU—clusterin concentration in µg/mL; SNA—relative reactivity of blood serum CLU glycans with *Sambucus nigra* agglutinin. SNA specificity is presented in Section 4.3. Relative reactivities of CLU glycans with lectin are expressed in absorbance units (AU). Here are presented only the results of the ROC curve analysis for which the AUC values were >0.700.

**Table 4:** The specificity of biotinylated lectins.

Name of Lectin	The Specificity of Lectin
SNA (*Sambucus nigra* agglutinin)	sialic acid α2,6-linked to antennary Gal [52]
MAA (*Maackia amurensis* agglutinin)	sialic acid α2,3-linked to antennary Gal [52]
LTA (*Lotus tetragonolobus* agglutinin)	antennary fucose α1,3-linked to GlcNAc [53]
UEA (*Ulex europaeus* agglutinin)	antennary fucose α1,2-linked to Gal and α1,3-linked to GlcNAc [54]
LCA (*Lens culinaris* agglutinin)	core fucose α1,6-linked [55]

Gal—galactose, GlcNAc—N-acetylglucosamine.

## Data Availability

All data needed to evaluate the conclusions in the article are present in the article. Additional data related to this study are available upon reasonable request from the corresponding author.
